# Mindsong – music therapy for dementia: music therapy during the Covid-19 pandemic

**DOI:** 10.1177/17579139211072377

**Published:** 2022-03-11

**Authors:** M Grady, R Melhuish

**Affiliations:** Director of Music Therapy, Mindsong, Gloucestershire, UK; Deputy Director of Music Therapy, Mindsong, Gloucestershire, UK

## Background

Mindsong is a charity specialising in supporting people with dementia and their carers through a range of music interventions based in Gloucestershire, in the UK.

This article focuses on our Music Therapy at Home (MT@H) service and describes how it has survived and thrived during the pandemic, illustrated with quotations from family carers.

Music Therapy is a clinical intervention provided by qualified, Health and Care Professions Council (HCPC)–registered therapists who aim to support emotional and psychological wellbeing for people with varying health conditions through music and song. Using techniques such as improvisation, songwriting, active listening or singing familiar songs, therapists engage with another person in a highly personalised way. For people with dementia, music therapy can achieve positive effects on communication, engagement, stimulation, mood, relationships and a sense of identity.^1^ A growing body of research suggests that it is an effective intervention for later-stages of dementia, supporting wellbeing and quality of life.^2–4^ Music Therapy is also recommended in the UK NICE Guidelines for dementia.^5^

Mindsong’s Music Therapy at Home service was conceived in 2016 as a co-production with our local NHS Clinical Commissioning Group (CCG), who continue to provide support. The service is for people living at home with later-stage or complex dementia who are being cared for by a family member. Our music therapists provide support for the family carer as well as the person with dementia, helping to reduce stress and improve quality of life for both.^1^ Part of the therapy is the development of personalised music strategies to support daily care and wellbeing outside session times, for example, during mealtimes or when completing personal care.^1,6^

We receive referrals from local Community Dementia Nurses, Later Life Mental Health Teams, GPs, Alzheimer’s Society Dementia Advisers and self-referrals. Despite the pandemic, in 2020, we saw our busiest year to date, working with 78 different families in Gloucestershire.

## The Intervention

Following their referral, the family carer is contacted by one of our Carer Supporters, all of whom have previous clinical experience in healthcare and receive ongoing training and support from Mindsong. They assess the situation by talking with the carer about the particular challenges and difficulties they face and completing a baseline evaluation form which measures the impact of caring on their daily life. This is called a Carer Dementia Quality of Life measure (C-DEMQoL^7^). The possible aims and outcomes of music therapy are discussed, and informed consent is gained from all parties. The carer may be asked to make a decision on behalf of the person with dementia.

**Figure:**
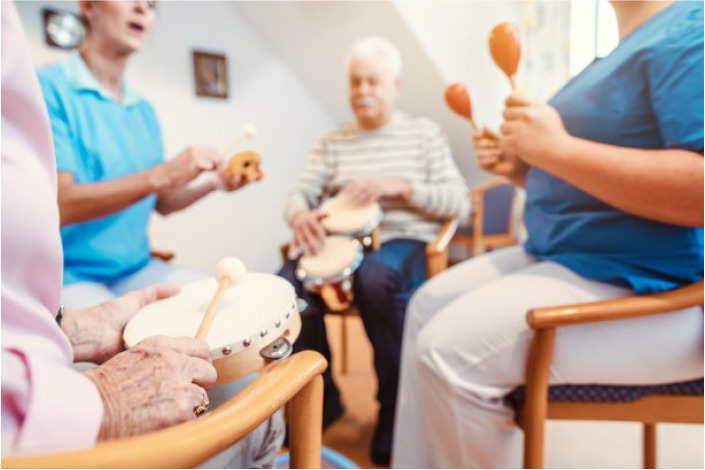


Once this has been agreed, Mindsong offers 12 weekly sessions in the family home, funded by Mindsong and Gloucestershire NHS CCG. Sessions usually last about an hour and are tailored to meet the couple’s needs. Sometimes, other family members or friends also join in.

## Impact of Covid-19 Pandemic

These have been extremely challenging times for people with dementia and their carers.^8–10^ Services such as respite care, community support groups and personal care were suddenly halted. Medical consultations were held online and in many cases, friends and family could no longer visit. Deterioration of care home residents due to social isolation has been widely reported in the press, but equally affected those with dementia living in the community. Family carers struggled with the extra pressures of trying to cope alone, and despite some improvements over the year as services began to adapt, for many families, the situation remains little changed.

Mindsong’s MT@H service has never been more essential, and we worked within restrictions to continue our work throughout the pandemic. As one carer observed,

*It was the first positive response from an organisation – others withdrew due to Covid.*

Initially offering therapy online or over the phone, in April 2020, we gained agreement from the local Dementia Commissioner and Gloucestershire Police for our therapists to travel and deliver Music Therapy outdoors. Sessions took place in people’s gardens, driveways or on front doorsteps, and this was greatly valued by carers:

*No-one else would come in … (the therapist) came and sat in the doorway.*

As autumn arrived, we moved inside, wearing personal protective equipment and adhering to strict safety guidelines to minimise the risk of transmission. We have continued indoor, outdoor and online sessions throughout the three lockdowns, providing much-needed social, psychological and practical support.

## Evaluation of Impact

Mindsong attaches great importance to understanding the impact of our services. Carer feedback is considered to be highly significant, and we continue to review our evaluation methods to improve our understanding of their experience and the outcomes of music therapy.

Our Carer Supporters play a crucial role in evaluating the therapy, conducting an interim phone call halfway through and a further evaluation when the 12 sessions are complete. This allows the carer to describe any effects of the music therapy, both for themselves and their relative.

Analysis of carer comments consistently shows benefits to communication and family relationships, mild to marked improvement in a variety of dementia symptoms and increased general wellbeing for all parties:

*Music lifts him to a time when problems didn’t exist.*

*Mindsong has been life-changing. It’s been absolutely brilliant for Mum and the family.*

Sometimes these benefits extend to wider family and social networks, leading to strengthened relationships, more support, and reduced isolation and loneliness. For instance, family and friends may visit more often when they find that singing is an easy and enjoyable way of reconnecting and spending time together. Feedback from our newly established online Carers’ Forum illustrates how the much support of Mindsong was valued:

*The feeling that ‘I mattered’ for the first time – that meant so much – it really carried me through those difficult times.*

For more information, please visit our website: mindsong.org.uk
